# Prognostically orientated multimodality treatment including surgery for selected patients of small-cell lung cancer patients stages IB to IIIB: long-term results of a phase II trial

**DOI:** 10.1038/sj.bjc.6690830

**Published:** 1999-12

**Authors:** W Eberhardt, G Stamatis, M Stuschke, H Wilke, M R Müller, S Kolks, M Flasshove, J Schütte, M Stahl, L Schlenger, V Budach, D Greschuchna, G Stüben, H Teschler, H Sack, S Seeber

**Affiliations:** Departments of Internal Medicine (Cancer Research) and,Radiotherapy, West German Cancer Center, University of Essen Medical School, Hufelandstraße 55, 45122 Essen, Germany; Department of Pneumology and Thoracic Surgery, Ruhrlandklinik, Essen-Heidhausen, Germany

**Keywords:** small-cell lung cancer, combined modality, surgery, neoadjuvant

## Abstract

Following mediastinoscopy, a prognostically orientated multimodality approach was chosen in selected small-cell lung cancer (SCLC) patients with hyperfractionated accelerated chemoradiotherapy (Hf-RTx) and definitive surgery (S). Stage IB/IIA patients had four cycles of cisplatin/etoposide (PE) and surgery. Stage IIB/IIIA patients had three cycles PE followed by one cycle concurrent chemoradiation including Hf-RTx and surgery. Most stage IIIB patients were not planned for surgery and had CTx followed by sequential RTx or one cycle concurrent CTx/RTx. Of 46 consecutive patients (stage IB six, IIA two, IIB/IIIA 22, IIIB 16) 43 (94%) showed an objective response. Twenty-three of patients (72%) planned for inclusion of S were completely resected (R0) (IB 6/6, IIA 2/2, IIB/IIIA 13/22, IIIB 2/2). Overall toxicity was acceptable – one patient died of septicaemia, no perioperative deaths occurred. Median follow-up of patients alive (*n* = 21) is 52 months (30+ – 75+). Median survival and 5-year survival rate of all patients are 36 months and 46%, in R0 patients 68 months and 63% (R0-IIB/IIIA/IIIB: not yet reached and 67%). This multimodality treatment including surgery proved highly effective with 100% local control and remarkable long-term survival after complete resection, even in locally advanced SCLC stages IIB/IIIA patients. © 1999 Cancer Research Campaign


*British Journal of Cancer* (1999) **81**(7), 1206–1212
© 1999 Cancer Research Campaign
Article no. bjoc.1999.0830

### Prognostically orientated multimodality treatment including surgery for selected patients of small-cell lung cancer patients stages IB to IIIB: long-term results of a phase II trial

**W Eberhardt[1], G Stamatis[3], M Stuschke[2], H Wilke[1], MR Müller[1], S Kolks[1], M Flasshove[1], J Schütte[1], M Stahl[1], L Schlenger[1], V Budach[2], D Greschuchna[3], G Stüben[2], H Teschler[3], H Sack[2] and S Seeber[1]**

Departments of [1]Internal Medicine (Cancer Research) and [2]Radiotherapy, West German Cancer Center, University of Essen Medical School; Hufelandstraße 55, 45122 Essen, Germany; [3]Department of Pneumology and Thoracic Surgery, Ruhrlandklinik, Essen-Heidhausen, Germany

**Summary** Following mediastinoscopy, a prognostically orientated multimodality approach was chosen in selected small-cell lung cancer (SCLC) patients with hyperfractionated accelerated chemoradiotherapy (Hf-RTx) and definitive surgery (S). Stage IB/IIA patients had four cycles of cisplatin/etoposide (PE) and surgery. Stage IIB/IIIA patients had three cycles PE followed by one cycle concurrent chemoradiation including Hf-RTx and surgery. Most stage IIIB patients were not planned for surgery and had CTx followed by sequential RTx or one cycle concurrent CTx/RTx. Of 46 consecutive patients (stage IB six, IIA two, IIB/IIIA 22, IIIB 16) 43 (94%) showed an objective response. Twenty-three of patients (72%) planned for inclusion of S were completely resected (R0) (IB 6/6, IIA 2/2, IIB/IIIA 13/22, IIIB 2/2). Overall toxicity was acceptable – one patient died of septicaemia, no perioperative deaths occurred. Median follow-up of patients alive ($n$ = 21) is 52 months (30+ – 75+). Median survival and 5-year survival rate of all patients are 36 months and 46%, in R0 patients 68 months and 63% (R0-IIB/IIIA/IIIB: not yet reached and 67%). This multimodality treatment including surgery proved highly effective with 100% local control and remarkable long-term survival after complete resection, even in locally advanced SCLC stages IIB/IIIA patients.
© 1999 Cancer Research Campaign

**Keywords**: small-cell lung cancer; combined modality; surgery; neoadjuvant

Small-cell lung cancer (SCLC) represents a distinct pathological and clinical entity, accounting for about 15–20% of all lung cancer cases (Parker et al, 1997). Due to its tendency to disseminate early, systemic combination chemotherapy has been the cornerstone of treatment and, depending on initial disease extent, between 80 and 100% of patients achieve objective responses (Ihde et al, 1997). In localized – limited – disease (LD-SCLC) patients can be cured and the rate of long-term survivors of 10–15% after 5 years can be increased to between 15 and 20% by early inclusion of thoracic irradiation (Pignon et al, 1992; Elias et al, 1997). However, even in modern chemoradiation protocols, most patients experience tumour recurrences both locally in the chest as well as at distant locations – preferably the brain (Arriagada et al, 1992, 1995). Recent strategies to improve outcome have included: (a) application of chemotherapy concurrently with radiation (Takeda et al, 1996); (b) intensification of radiotherapy (hyperfractionated accelerated radiation) (Turrisi et al, 1992); (c) combination of both (a) and (b) with integration of radiation as early as possible; and (d) increase of chemotherapy dose intensities supported by autologous bone marrow or peripheral stem cell transplantation (Elias et al, 1993). However, both strategies – to increase local control by aggressive radiation techniques – as well as an escalation of systemic treatment intensity – have been hampered by a high rate of local failures between 30 and 50% (Gray et al, 1995; Elias et al, 1997; Turrisi et al, 1999). Similar to other solid tumours, surgery may represent the most effective local treatment for dealing with residual disease at the bulky primary tumour, even though we do not know which patient subgroups will eventually profit concerning local control, survival, long-term survival or even cure (Choi et al, 1997). On this background, we started a phase II trial in selected SCLC patients, mostly stages IIB, IIIA and IIIB surgically staged by mediastinoscopy, in whom best results in recent concurrent chemoradiation protocols have been reported with 15–26% 5-year survival rates (McCracken et al, 1990; Turrisi et al, 1999). Aim of the study was to evaluate feasibility, toxicity and efficacy of a prognostically orientated approach in patients consecutively conferred to our institution. Treatment included upfront cisplatin-based chemotherapy, early concurrent chemoradiation with hyperfractionated accelerated radiotherapy in locally advanced/mediastinal risk stages (IIB/IIIA), as well as definitive surgery, if possible, adding up to an aggressive trimodality treatment for the majority of patients.

*Received 2 November 1998*
*Revised 28 April 1999*
*Accepted 7 June 1999*

*Correspondence to:* W Eberhardt. E-mail: Wilfried.eberhardt@uni-essen.de

### PATIENTS AND METHODS

### Patient selection

Patients with histologically/cytologically proven SCLC were eligible. Following mediastinoscopy patients with SCLC stages IB–IIIB were taken onto this trial (Mountain et al, 1997). No patient with T1NO (IA) was included. Furthermore, patients with malignant pleural effusion or with ipsi- or contralateral supraclavicular lymph node involvement were excluded. Further eligibility criteria were performance status (WHO) of less than 2, age between 18 and 70 years, no prior treatment and no other malignancy.

Following a complete medical history patients underwent a general physical examination. A complete blood cell count, serum chemistry, coagulation tests and urinalysis were performed. Staging included chest radiographs, computerized tomography (CT) scans of the chest, upper abdomen and brain, abdominal ultrasound, radionuclide bone scan, bronchoscopy with biopsies of prospective resection margins and (cervical) mediastinoscopy with multiple biopsies of paratracheal, tracheobronchial, subcarinal nodes and other suspicious mediastinal structures. In case of suspected pulmonary artery invasion, an angiographic CT scan of the thorax was performed.

Patients were evaluated in an interdisciplinary conference, and this risk analysis was repeated preoperatively after restaging and included cardiopulmonary function tests (Eberhardt et al, 1998). Characteristics that rendered patients ineligible/functionally inoperable were a prognosticated post-operative FEV1 of less than 1 litre (Eberhardt et al, 1998). All patients were required to have normal haematological parameters and adequate organ functions. Patients with serious concomitant infections were excluded. All patients were fully informed about the nature and purpose of this study and gave informed consent. The study protocol had approval by the local ethics committee.

### Prognostically orientated treatment

Eligible patients with stages IB/IIA had four cycles cisplatin/etoposide (PE), and after the fourth cycle thoracotomy was planned (Figure 1). Stage IIB/IIIA patients were given three cycles PE and, during week 10, concurrent chemoradiotherapy was started combining twice-daily hyperfractionated accelerated radiotherapy with one cycle of PE. Following this, repeat mediastinoscopy was performed in patients with initially involved mediastinum/mediastinal nodes, and surgery followed 3–5 weeks after the end of radiation. Patients with N3-disease at mediastinoscopy or T4-disease in angiographic CT scans of the chest (IIIB), were generally not planned for a preoperative approach but

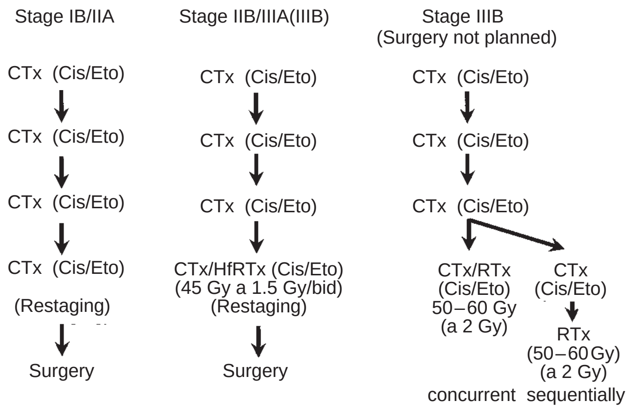

**Figure 1** Prognostically orientated treatment schema. Ctx = chemotherapy; HfRTx = hyperfractionated accelerated radiotherapy; Rtx = radiotherapy; Cis = cisplatin; Eto = etoposide received four chemotherapy cycles and sequential radiation up to 50 Gy in 2-Gy daily fractions. Towards the end of the study period, patients had the fourth cycle given concurrently to radiation (2 Gy daily fractions up to between 50 and 60 Gy). During the last year of the study, two patients with stage IIIB were planned for a preoperative approach.

### Induction chemotherapy

Chemotherapy consisted of cisplatin, 50 mg $m^{-2}$ as 1-h infusion on days 1 and 7 (or 8 if outpatient) (Wilke et al, 1988). Prior to cisplatin, patients had adequate hydration with 1000 ml of 0.9% normal saline, and diuresis was started with 40 mg furosemide. Cisplatin was given in 1000 ml 0.9% normal saline. After cisplatin, patients received 2000 ml 0.9% normal saline together with 30 mEq magnesium chloride over 2 h. Adequate anti-emetics (decadronphosphate i.v./ondansetron i.v.) were given with cisplatin and on the following day. Etoposide was given at 170 mg $m^{-2}$ in 500 ml 0.9% normal saline as 1-h infusion on days 3, 4 and 5. The majority of patients was hospitalized for chemotherapy. Administration of the following cycle on day 22 was postponed if patients had a white blood cell (WBC) count < 2500 $l^{-1}$ or a platelet count < 100 000 $l^{-1}$ until these values were reached. Dose reductions were performed as described for our NSCLC-preoperative trial (Eberhardt et al, 1998). In none of the patients included chemotherapy was supported with haematopoietic growth factors.

### Preoperative chemoradiotherapy

A total dose of 45 Gy was given to the primary and the mediastinal nodes in two daily fractions of 1.5 Gy within 3 weeks. The minimum time interval between fractions was 6 h. The technique of radiation delivery was the same as in our NSCLC-trimodality trial (Eberhardt et al, 1998). Simultaneous chemotherapy was started on day 2 of radiation (cisplatin 50 mg $m^{-2}$ days 2 and 9, etoposide 100 mg $m^{-2}$ days 4, 5 and 6).

### Definitive radiation/chemoradiation

Patients with stage IIIB were generally not planned for inclusion of surgery (Figure 1). Patients during the first year of the study received four cycles PE and had radiation therapy sequentially with conventionally fractionated radiation at 2 Gy per day up to 50 Gy. All following patients had radiation given concurrently to the fourth chemotherapy cycle. Radiotherapy was fractionated at 2 Gy per day up to between 50 and 60 Gy.

### Prophylactic cranial irradiation

Routinely, prophylactic cranial irradiation (PCI) was offered. PCI was started after the end of the fourth chemotherapy cycle on day 9 of thoracic irradiation in patients with chemoradiation, or was given following thoracotomy in patients with stages IB and IIA. A total dose of 30 Gy in 3 weeks was given in daily fractions of 2 Gy.

### Repeat mediastinoscopy/surgery

N2-patients were planned for a repeat mediastinal exploration, including biopsies of all initially involved mediastinal structures/ nodes. Only patients with negative nodes following induction

*© 1999 Cancer Research Campaign*

Table 1 Patient characteristics

| Characteristic | No. | % |
|---|---|---|
| All patients | 46 | 100 |
| Stage | | |
| IB T2N0 | 6 | 13 |
| IIA T2N1 | 2 | 4 |
| IIB T3N0 | 4 | 9 |
| IIIA | 18 | 39 |
| T3N1 | 2 | |
| T2N2 | 10 | |
| T3N2 | 6 | |
| IIIB | 16 | 35 |
| T4N0/N1 | 3 | |
| T4N2 | 3 | |
| T2N3 | 3 | |
| T3N3 | 3 | |
| T4N3 | 4 | |
| Age, years | | |
| Median | 55 | |
| Range | 34–69 | |
| Sex | | |
| Male | 32 | 70 |
| Female | 14 | 30 |
| Performance status, WHO | | |
| Median | 0 | |
| 0 | 35 | 76 |
| 1 | 11 | 24 |
| Histological cell type | | |
| Small-cell carcinoma | 43 | 94 |
| Mixed small-cell/large-cell | 3 | 6 |
| Lactate dehydrogenase Median (U/l) | 213 | |
| Range (U/l) | 110–393 | |

proceeded to surgery, whereas boost irradiation up to 60 Gy was offered to patients with persistent mediastinal disease. Three to 5 weeks after end of irradiation, surgery was planned. Surgical procedures included lobectomies, bilobectomies, sleeve resections, chest wall resections or pneumonectomies as indicated. Because our NSCLC study had shown an increased incidence of stump insufficiencies, bronchial anastomoses were routinely protected with a flap of intercostal muscle in situations considered with an increased risk.

### Response and toxicity evaluation

Responses were assessed using standard WHO criteria (Miller et al, 1981). After surgery, patients were staged using UICC criteria. Toxicities were assessed using the WHO criteria, the RTOG acute radiation toxicity criteria and the RTOG/European Organization for Research and Treatment of Cancer (EORTC) late radiation toxicity criteria (Miller et al, 1981).

### Statistics

Survival was measured from first day of chemotherapy until death, loss to follow-up or time of evaluation for this report. Event-free survival was calculated from first day of chemotherapy until any event occurring such as tumour progression, incidence of second cancer, death due to toxicity, secondary conditions or second malignancy (Green et al, 1994). Survival curves were estimated by the method of Kaplan and Meier (1958). Differences in curves between groups of patients were evaluated using a log-rank test (Mantel et al, 1966).

### RESULTS

### Patient characteristics

Forty-six consecutive SCLC patients were enrolled at our institution from June 1991 until July 1995 and all are eligible for this report. Patient characteristics are given in Table 1. The four stage IIB patients had large centrally located bulky T3N0-tumours with invasion of the mediastinal/pericardial pleura. There were 19 patients with positive N2- and ten with N3-lymph nodes, adding up to 29 of 46 patients (63%) with proven mediastinal node disease. Among the patients with IIIB-disease, ten had T4-disease, six pulmonary artery invasion and four diffuse mediastinal organ infiltration.

### Induction chemotherapy

All 46 patients received at least one chemotherapy cycle. One patient died during the first cycle due to septicaemia and was not evaluable for response. One patient with a cerebral infarction during the first cycle and one patient with an infection after the second cycle had to be taken off study. All remaining 43 patients received at least three cycles. Altogether, 134 chemotherapy cycles have been applicated and only in 16 cycles treatment had to be postponed for less than 1 week with dose reduction in ten cycles. All eight patients with stage IB/IIA had the four planned cycles and thoracotomy performed.

### Induction chemoradiotherapy

All 22 patients with stage IIB/IIIA and two further patients with stage IIIB were planned for a preoperative chemoradiotherapy induction. Five patients, one due to decline in performance status and four due to medical reasons, did not proceed to thoracotomy. Two further patients refused surgery and received boost irradiation up to 60 Gy. Thus, of overall 24 patients initially planned to receive the complete preoperative chemoradiation block, this could be delivered in 22. No interruptions in radiation delivery had to be made.

### Definitive chemoradiotherapy

Of 16 patients with stage IIIB, 14 were initially *not* planned for inclusion of surgery. In the first year of the study, four were treated with four cycles PE and received sequential radiation up to 50 Gy. Nine of the ten following patients were given concurrrent chemoradiation with one cycle simultaneously to radiation (50–60 Gy).

### Maximum clinical response

The maximum clinical response to induction was evaluable in 45 patients. Sixteen clinical complete responses (CR) (35%) and 27 partial remissions (PR) (59%) were achieved, adding up to 43 objective responses (94%). Stable disease (NC) was found in two patients (4%). No patient showed early progression. Most patients had continuously shrinking tumours after each cycle with maximum response following the second cycle and ten patients with further major improvement to chemoradiotherapy (seven PR converted to CR, three NC converted to PR). There were no significant differences in response rates and rates of stable disease between TNM-subgroups or disease stages.

 © 1999 Cancer Research Campaign

**Table 2** Eligibility for thoracotomy and surgical outcome

| Patient group | No. | % |
|---|---|---|
| All patients | 46 | |
| Not planned for surgery (IIIB, CTx+RTx or CTx/RTx) | 14 | |
| Planned for surgery (stages IB–IIIB) | 32 | 100 |
| Not operable (medical, refusal) | 8 | |
| Operable | 24 | 24/32 75% |
| Completely resected (R0) | 23 | 23/32 72% |
| Resection of vital tumour tissue | 12 | 12/32 38% |
| Macroscopic tumour | 7 | |
| Microscopic tumour | 5 | |
| Small-cell histology | 9 | |
| Non-small-cell histology | 3 | |
| Pathological complete response (pCR) | 11 | 11/32 34% |

### Eligibility for thoracotomy and surgical outcome

Thirty-two patients – all of stages IB–IIIA and two of stage IIIB – were planned for inclusion of surgery. In 16 patients with involved mediastinal nodes, repeat mediastinoscopy was performed before consideration of surgery (14 in stage IIIA and two in IIIB). In three patients with stage IIIA, positive N2-nodes were found and surgery was no longer found appropriate. Thus, 13 of 16 patients (81%) with initially positive mediastinal nodes had an N0-status after the induction. No patient with N0 at repeat mediastinoscopy had microscopic N2-status in his final pathology. At the end of induction chemotherapy±chemoradiation, 24 of the planned 32 patients (75%) could be operated on (Table 2). One patient turned out irresectable with multiple pleural metastases. In all remaining 23 (72% of patients planned for surgery) the primary tumour could be completely resected. Surgical procedures were: ten pneumonectomies, two bilobectomies and 11 lobectomies. Of these 23 completely resected patients, 12 (52%) had viable tumour tissue (five with microscopic, seven with macroscopic tumour). Histological examinations revealed nine patients with vital pure small-cell carcinoma and three with non-small-cell pathology – two with squamous cell and one with large-cell anaplastic carcinoma. 11 patients (34% of those eligible for surgery) had no vital tumour in resected specimens (pathological complete response – pCR) (Table 2).

### Toxicities

Maximum toxicity in all 46 patients during induction is listed in Table 3. Grade 4 haematologic toxicity occurred in 12 (26%). Severe oesophagitis (grade 3) occurred in five patients, but was usually of short duration (< 10 days). Three required intravenous hydration with grade 4 oesophagitis, but radiation was not interrupted and oesophagitis completely recovered within 1 week. No oesophageal stricture was observed. One patient died due to septicaemia during the first chemotherapy cycle. Additional grade 3 toxicities were hearing loss (one patient), peripheral neuropathy (one patient) and renal impairment (one patient). No peri- or post-operative deaths occurred. No stump insufficiency or pleural empyema was observed. Four patients received platelet transfusions due to serious thrombocytopenia, six patients received erythrocyte transfusions due to symptomatic anaemia and three had to be taken on antibiotics for fever and infection. Pneumonitis of grade 3 was seen in two patients. To summarize, only one patient (2%) died from causes that could be directly attributed to this intensive multimodality treatment.

**Table 3** Maximum toxicity during chemotherapy ± chemoradiotherapy

| | WHO grade (*n* = 46) | | | | |
|---|---|---|---|---|---|
| Toxicity | 1 | 2 | 3 | 4 | 5 |
| Haematological | | | | | |
| Leukocytopenia | 6 | 11 | 22 | 6 | 0 |
| Thrombocytopenia | 6 | 16 | 10 | 5 | 0 |
| Anaemia | 11 | 24 | 8 | 1 | 0 |
| Non-haematological | | | | | |
| Fever/infection | 4 | 5 | 3 | 0 | 1 |
| Nausea and vomiting | 12 | 27 | 3 | 0 | 0 |
| Neurological | 39 | 5 | 1 | 0 | 0 |
| Oesophagus | 17 | 14 | 5 | 3 | 0 |
| Lung | 19 | 9 | 2 | 0 | 0 |
| Heart | 8 | 2 | 1 | 0 | 0 |
| Ototoxicity | 1 | 0 | 1 | 0 | 0 |
| Renal | 12 | 2 | 1 | 0 | 0 |
| Other | 12 | 6 | 0 | 0 | 0 |
| Worst effect reported per patient | 2 | 12 | 23 | 8 | 1 |

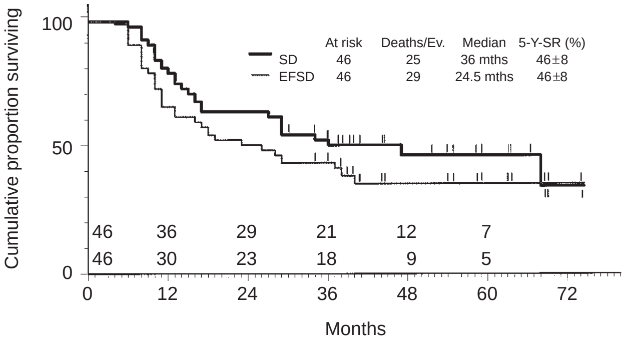

**Figure 2** Survival durations (SD) and event-free survival durations (EFSD) in all patients. SD = survival duration; EFSD = event-free survival duration; mths = months; 5-Y-SR = 5-year survival rate

### Survival

The median follow-up for all patients was 53 months and 52 for those alive at the time of this report (assessed 1 December 1997). Median survival for all 46 eligible patients was 36 months and actuarial 5-year survival was 46% (Figure 2). Median event-free survival was 24.5 months with 5-year event-free survival of 36%. The last events at 37, 38 and 40 months were second respiratory cancers. No SCLC-recurrence was seen after 29 months. Of six patients with stage IB, three remain alive at 54, 63 and 66 months. Of two patients with stage IIA, one is alive at 63 months. In 22 patients with stage IIB/IIIA, median survival has not yet been reached with 5-year survival of 50% and 11 patients alive from 36 to 75 months. In 16 patients with stage IIIB, median survival was 29 months and 5-year survival 35%. Six remain alive at 30–69 months. No significant difference in survival has been observed between stages IB/IIA, IIB/IIIA and IIIB. Median event-free survival has been 29 months for stage IIIB, not yet reached in IIA, 33.5 months for IIB/IIIA and 15 months in IIIB (IIB/IIIA versus IIIB: $P = 0.13$). No differences in survival and event-free survival have been observed between different TNM-categories or subgroups defined by N- and T-parameters, age, sex and initial LDH-values. CR-patients have a median survival of 28 months (30% actuarial 5-year survival). Patients with PR have not yet

© 1999 Cancer Research Campaign

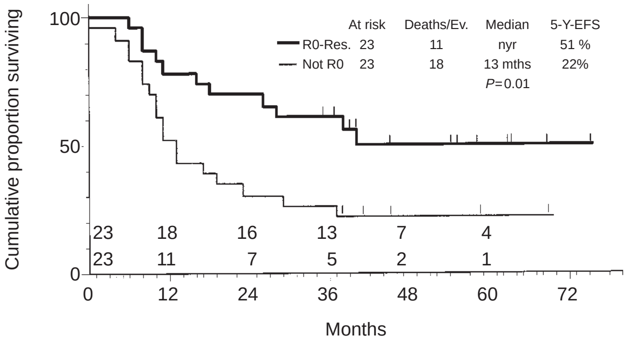

**Figure 3** Event-free survival duration in completely resected patients and patients not completely resected – stages IB–IIIB. R0-Res. = R0-(complete) resection; mths = months; 5-Y-EFS = 5-year event-free survival; nyr = not yet reached

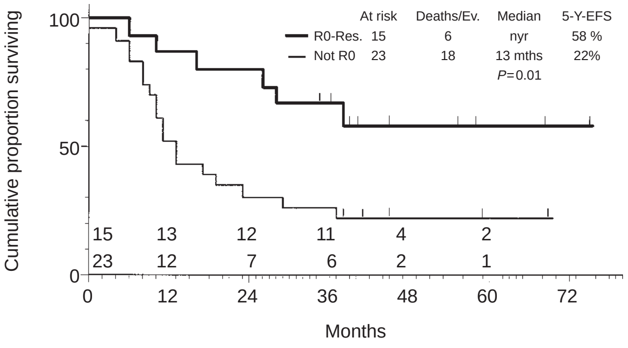

**Figure 4** Event-free survival durations in completely resected patients and patients without complete resection – stages IIB/IIIA/IIIB only. R0-Res = R0-(complete)resection; mths = months; 5-Y-EFS = five-year event-free survival; nyr = not yet reached reached median survival (59% at 34 months). Of two NC patients one remains alive following R0-resection at 40 months.

### Outcome of patients with complete resection (RO)

Patients with complete resection (R0) had a median survival of 68 months and a 5-year survival of 63%. Patients in whom complete resection was not part of treatment, had a significantly shorter median survival of 17 months and a 5-year survival of 30% ($P$ = 0.01). Among patients with R0-resection, no differences in long-term survival were found between those with pCR and those with persistent viable tumour (5-year-survival rate 73% vs 57%, $P$ = 0.75). Actuarial event-free survival has stabilized at 51% after 5 years for all R0-patients and 58% after 5 years for R0-patients of stages IIB, IIIA and IIIB (Figures 3 and 4). Of these completely resected patients 12 remain alive and event-free between 34 and 75 months.

### Relapse pattern

Of the 23 R0-patients, nine (36%) have relapsed. The only site of relapse was distant metastases: eight with isolated central nervous system (CNS) relapse and one with liver metastases. No patient experienced local/locoregional failure. Two patients developed second primaries – one in the contralateral middle lobe and one tracheal carcinoma. Locoregional relapse was seen if complete resection was not part of treatment. Six of 23 patients (26%) in this subgroup experienced locoregional failure as first site of relapse, four of these as the only location of failure. Twelve of 23 patients (52%) developed distant metastases as first relapse, only in two patients simultaneously to locoregional failure. Again, the brain was the most frequent relapse site, with seven of 23 patients (30%) as first site of failure. One patient in this cohort experienced a second primary in the respiratory tract (non-small-cell lung cancer).

### DISCUSSION

The present trial investigated feasibility, toxicity and efficacy of a prognostically orientated multimodality treatment including surgery for selected SCLC-patients. Entered were stages IB–IIIB, without malignant effusions or supraclavicular node involvement, although the majority presented with bulky tumours and proven mediastinal disease at staging mediastinoscopy. Our results showed high resectability rates in stages IB–IIIA after chemotherapy plus/minus chemoradiotherapy. No major perioperative morbidity nor any post-operative mortality was observed that may be attributed to a consequent protection of bronchial anastomosis intraoperatively (Eberhardt et al, 1998). With mature long-term follow-up, the median survival for the whole patient cohort of 36 months and an actuarial survival rate of 46% at 5 years are clearly encouraging.

So far, the best results for SCLC patients have been reported from combined (concurrent) chemoradiation protocols with median survival between 18 and 25 months and 5-year survival of 15–25%, but these were groups with limited disease, not generally excluding positive pleural effusions or involved supraclavicular nodes (McCracken et al, 1990; Turrisi et al, 1992). Twice daily thoracic radiotherapy – included early in the treatment – has been convincingly demonstrated to have an effect on both local control and long-term survival (Turrisi et al, 1999). A different approach intensifies the systemic component (chemotherapeutic dose-intensities) supported by autologous bone marrow or peripheral stem cell transplantation (Elias et al, 1997; Fetscher et al, 1997). A small pilot trial in selected patients reported a median survival of 29 months and 5-year survival of 52%, but also observed a remarkably high local-failure rate (Elias et al, 1993). However, high-dose treatment may not be possible in most elderly lung cancer patients and can lead to increased toxicities with mortality rates reported up to 13% (Fetscher et al, 1997).

A major systemic risk for SCLC patients with prolonged survival duration has turned out to be development of brain metastases. A carefully performed meta-analysis of published randomized trials demonstrated a significant effect of prophylactic cranial irradiation (PCI) on survival in patients with complete response (Arriagada et al, 1998).

In contrast, insufficient local control at bulky tumour areas can be another reason for eventual recurrence. Even after three to four chemotherapy cycles a rate of vital residual disease at the primary site between 55 and 80% has been found at adjuvant surgery (Shepherd et al, 1996). Interestingly, some of the resected specimens demonstrate a change to NSCLC-histopathologies – whether treatment induced or simply a result of initial tumour cell heterogeneity is still not clear (Shepherd et al, 1988). Some investigators

© 1999 Cancer Research Campaign have given chemotherapy preoperatively to stages I–IIIA (Shepherd et al, 1989; Zatopek et al, 1991; Müller et al, 1992), others post-operatively (Macchiarini et al, 1989; Ulsperger et al, 1991; Davis et al, 1993). Modern cisplatin-based chemotherapy was rarely used and different policies concerning integration of radiotherapy were chosen. Only one study has given a bimodal induction combining chemotherapy and radiotherapy, but the patient number finally operated on was too small to draw conclusions (Gridelli et al, 1994).

The only randomized trial testing surgery following induction chemotherapy came from the Lung Cancer Study Group (LCSG). After five chemotherapy cycles, responding patients were randomized to receive either chest radiotherapy or surgery followed by radiotherapy (Lad et al, 1994). Preliminary results of this trial did not support the use of surgery in comparable patient subgroups. However, the patient selection of the LCSG-trial was not comparable to the one in our study. The LCSG did not include staging mediastinoscopy, patients were not a priori assessed to be resectable and were randomized only following response to chemotherapy. Moreover, this trial has also been criticized concerning its final conclusions and consequences (Shepherd et al, 1996).

On this background, we reconsidered surgery as definitive local treatment following induction chemotherapy plus/minus concurrent chemoradiotherapy in stages IB–IIIA. Of note is the high rate of 100% locoregional control following complete resection. Median survival of 68 months and a 5-year survival of 63% in this subgroup are promising and point to the efficacy of this approach also in patients in stage IIIA (N2). Different to our findings with preoperative chemoradiation, earlier investigations with chemotherapy alone followed by surgery had been disappointing for mediastinal N2-disease (IIIA(N2)) (Meyer et al, 1984; Shepherd et al, 1993).

In conclusion, surgery in SCLC can be considered in stages IB and IIA following chemotherapy, but has also demonstrated to be feasible in stages IIB and IIIA following induction chemoradiotherapy. Its definitive value on local control or long-term prognosis could only be evaluated in a further randomized trial that is urgently needed in this disease with overall poor long-term results and in which the impact of local control may have been underestimated.

### ACKNOWLEDGEMENTS

We express our deepest gratitude to all the nurses, physicians, and others who provided care and support for the patients who participated in this study.

### REFERENCES

Arriagada R, Kramar A, Le Chevalier T and De Cremoux H (1992) Competing events determining relapse-free survival in limited small-cell lung carcinoma. *J Clin Oncol* **10**: 447–451

Arriagada R, Le Chevalier T, Borie F, Rivière A, Chomy P, Monnet I, Tardivon A, Viader F, Tarayre M and Benhamou S (1995) Prophylactic cranial irradiation for patients with small-cell lung cancer in complete remission. *J Natl Cancer Inst* **87**: 183–190

Arriagada R, Auperin A, Pignon JP, Gregor A, Stephens R, Kristjansen PEG, Johnson B, Ueoka H, Wagner H and Whitacre M (1998) Prophylactic cranial irradiation overview in patients with small-cell lung cancer in complete remission. *Proc Am Soc Clin Oncol* **17**: 457 (abstr 1758)

Choi NC, Carey RW, Daly W, Mathisen D, Wain J, Wright C, Lynch T, Grossbard M and Grillo H (1997) Potential impact on survival of improved tumor downstaging and resection rate by preoperative twice-daily radiation and concurrent chemotherapy in stage IIIA non-small-cell lung cancer. *J Clin Oncol* **15**: 712–722

Davis S, Crino L, Tonato M, Darwish S, Pelicci PG and Grignani F (1993) A prospective analysis of chemotherapy following surgical resection of clinical stage I–II small cell lung cancer. *Am J Clin Oncol* **16**: 93–95

Eberhardt W, Wilke H, Stamatis G, Stuschke M, Harstrick A, Menker H, Krause B, Müller MR, Stahl M, Flasshove M, Budach V, Greschuchna D, Konietzko N, Sack H and Seeber S (1998) Preoperative chemotherapy followed by concurrent chemoradiation therapy based upon hyperfractionated accelerated radiotherapy and definitive surgery in locally advanced non-small-cell lung cancer: mature results of a phase II trial. *J Clin Oncol* **16**: 622–634

Elias AD, Ayash L, Frei III E, Skarin AT, Hunt M, Wheeler C, Schwartz G, Mazanet R, Tepler I, Eder JP, McCauley M, Herman T, Schnipper L and Antman KH (1993) Intensive combined modality therapy for limited-stage small-cell lung cancer. *J Natl Cancer Inst* **85**: 559–566

Elias AD (1997*a*) Small cell lung cancer: state-of-the-art therapy in 1996. *Chest* **112**: 251S–258S

Elias AD (1997*b*) Dose-intensive therapy in lung cancer. *Cancer Chemother Pharmacol* **40**: S64–S69

Fetscher S, Brugger W, Engelhardt R, Kanz L, Hasse J, Frommhold H, Wenger M, Lange W and Mertelsmann R (1997) Dose-intense therapy with etoposide, ifosfamide, cisplatin, and epirubicin (VIP-E) in 100 consecutive patients with limited- and extensive-disease small-cell lung cancer. *Ann Oncol* **8**: 49–56

Gray JR, Sobczak ML, Hahn SM, Sullivan FJ, Johnson BE and Bridges JD (1995) Analysis of local control in 150 limited stage small-cell-lung cancer patients treated with combined thoracic irradiation and multiagent chemotherapy. *Proc Am Soc Clin Oncol* **14**: 349 (abstr 1056)

Green MR, Cox JD, Ardizzoni A, Arriagada R, Bureau G, Darwish S, Deneffe G, Fukuoka M, Joseph D and Komaki R (1994) Endpoints for multimodal clinical trials in stage III non-small cell lung cancer (NSCLC): a consensus report. *Lung Cancer* **11** Suppl 3: S11–S13

Gridelli C, D'Aprile M, Curcio C, Brancaccio L, Palmeri S, Comella G, Veltri E, Ferrante G, Gentile M and Rossi A (1994) Carboplatin plus epirubicin plus VP-16, concurrent 'split course' radiotherapy, and adjuvant surgery for limited small-cell lung cancer. *Lung Cancer* **11**: 83–91

Ihde DC, Pass HI and Glatstein EJ (1997) Small cell lung cancer. In: *Cancer: Principles and Practice of Oncology*, 5th edn, DeVita VT Jr, Hellman S and Rosenberg SA (eds), pp. 911–949. Lippincott: Philadelphia

Johnson DH, Kim K, Sause W, Komaki R, Wagner H, Aisner S, Livingston R, Blum R and Turrisi AT (1996) Cisplatin and etoposide plus thoracic radiotherapy administered once or twice daily in limited stage small cell lung cancer: Final report of Intergroup Trial 0096. *Proc Am Soc Clin Oncol* **15**: 374 (abstr 1113)

Kaplan EL and Meier P (1958) Nonparametric estimation from incomplete observations. *J Am Statist Assoc* **53**: 457–481

Lad T, Piantadosi S, Thomas P, Payne D, Ruckdeschel J and Giaccone G (1994) A prospective randomized trial to determine the benefit of surgical resection of residual disease following response of small-cell lung cancer to combination chemotherapy. *Chest* **106**: 320S–323S

McCracken JD, Janaki LM, Crowley JJ, Taylor SA, Shankir Giri PG, Weiss GB, Gordon W, Baker LH, Mansouri A and Kuebler JP (1990) Concurrent chemotherapy/radiotherapy for limited small-cell lung carcinoma: a Southwest Oncology Group study. *J Clin Oncol* **8**: 892–898

Macchiarini P, Mussi A, Basolo F, Bruno J and Angeletti CA (1989) Optimal treatment for T1-3N0M0 small cell lung cancer: surgery plus adjuvant chemotherapy. *Anticancer Res* **9**: 1623–1625

Mantel N (1966) Evaluation of survival data and two new rank order statistics arising in its consideration. *Cancer Chemother Rep* **50**: 163–170

Meyer JA, Gullo JJ, Ikins PM, Comis RL, Burke WA, Di Fino SM and Parker FB Jr (1984) Adverse prognostic effect of N2 disease in treated small-cell carcinoma of the lung. *J Thorac Cardiovasc Surg* **88**: 495–501

Miller AB, Hoogstraten B, Staquet M and Winkler A (1981) Reporting results of cancer treatment. *Cancer* **47**: 207–214

Mountain CF (1997) Revisions in the international system staging lung cancer. *Chest* **111**: 1710–1717

Müller LC, Salzer GM, Huber H, Prior C, Ebner I, Frommhold H and Präuer HW (1992) Multimodal therapy of small cell lung cancer in TNM stages I through IIIa. *Ann Thorac Surg* **54**: 493–497

Parker SL, Tong T, Bolden S and Wingo PA (1997) Cancer statistics, 1997. CA Cancer J Clin **47**: 5–27

Pignon JP, Arriagada R, Ihde DC, Johnson DH, Perry MC, Souhami RL, Brodin O, Joss RA, Kies MS, Lebeau B, Onoshi T, Osterlind K, Tattersall MHN and Wagner H (1992) A meta-analysis of thoracic radiotherapy for small-cell lung cancer. *N Engl J Med* **327**: 1618–1624

*© 1999 Cancer Research Campaign* 

Shepherd FA, Evans WK, Feld R, Young V, Patterson GA, Ginsberg R and Johansen E (1988) Adjuvant chemotherapy following surgical resection for small-cell carcinoma of the lung. *J Clin Oncol* **6**: 832–838

Shepherd FA, Ginsberg RJ, Patterson GA, Evans WK and Feld R (1989) A prospective study of adjuvant surgical resection after chemotherapy for limited small cell lung cancer. A University of Toronto Lung Oncology Group study. *J Thorac Cardiovasc Surg* **97**: 177–186

Shepherd FA, Ginsberg RJ, Haddad R, Feld R, Sagman U, Evans WK, De Boer G and Maki E (1993) Importance of clinical staging in limited small-cell lung cancer: a valuable system to separate prognostic subgroups. *J Clin Oncol* **11**: 1592–1597

Shepherd F (1996) Surgical management of small-cell lung cancer. In: *Lung Cancer: Principles and Practice*. Pass HI, Mitchell JB, Johnson DH and Turrisi AT (eds), pp. 889–913. Lippincott-Raven: Philadelphia

Takada M, Fukuoka M, Furuse K, Ariyoshi Y, Ikegami H, Kurita Y, Nishiwaki Y, Nishiwaki H, Watanabe K, Noda K and Saijo N (1996) Phase III study of concurrent versus sequential thoracic radiotherapy in combination with cisplatin and etoposide for limited-stage small cell lung cancer: Preliminary results of the Japan Clinical Oncology Group. *Proc Am Soc Clin Oncol* **15**: 372 (abstract 1103)

Turrisi AT, Glover DJ, Mason B and Tester W (1992) Long term results of platinum etoposide + twice-daily thoracic radiotherapy for limited small-cell lung cancer: results on 32 patients with 48 months minimum F/U. *Proc Am Soc Clin Oncol* **11**: 292 (abstract 975)

Turrisi AT, Kim K, Blum R, Sause WT, Livingston RB, Komaki R, Wagner H, Aisner S and Johnson DH. Twice-daily compared with once-daily thoracic radiotherapy in limited small-cell lung cancer treated concurrently with cisplatin and etoposide. *N Engl J Med* **340**: 265–271

Ulsperger E, Karrer K and Denck H (1991) Multimodality treatment for small cell lung cancer. Preliminary results of a prospective, multicenter trial. The ISC-Lung Cancer Study Group. *Eur J Cardiothorac Surg* **5**: 306–310

Wilke H, Achterrath W, Schmoll HJ, Gunzer U, Preusser P and Lenaz L (1988) Etoposide and split-dose cisplatin in small cell lung cancer. *Am J Clin Oncol* **11**: 572–578

Zatopek NK, Holoye PY, Ellerbroek NA, Hong WK, Roth JA, Ryan MB, Komaki R, Pang AC and Glisson BS (1991) Resectability of small cell lung cancer following induction chemotherapy in patients with limited disease (stage II–IIIb). *Am J Clin Oncol* **14**: 427–432

 *© 1999 Cancer Research Campaign*
